# Hepatitis B Virus X Protein Upregulates SREBP2 to Modulate Autophagy in Hepatocellular Carcinoma

**DOI:** 10.1002/cam4.70916

**Published:** 2025-06-06

**Authors:** Qiuyan Lin, Yongxu Lin, Yongzhu Huang, Mingrong Wang, Xiaoxia Xie, Weiqi Cai, Qilan Guo, Pingying Jiang, Yuanlin Qi, Dan Li

**Affiliations:** ^1^ Department of Gastroenterology and Fujian Institute of Digestive Disease Fujian Medical University Union Hospital Fuzhou Fujian China; ^2^ Fujian Clinical Research Center for Digestive System Tumors and Upper Gastrointestinal Diseases Fuzhou Fujian China; ^3^ Department of Infection Fujian Provincial Hospital Fuzhou Fujian China; ^4^ School of Basic Medical Sciences Fujian Medical University Fuzhou Fujian China

**Keywords:** autophagy, cholesterol, Hepatitis B virus X protein, hepatocellular carcinoma, sterol regulatory element binding protein 2

## Abstract

**Background:**

The interaction between Hepatitis B virus X protein (HBx) and sterol regulatory element binding protein 2 (SREBP2) in modulating autophagy to influence inflammation and tumorigenesis is not fully understood. This research seeks to clarify the regulatory role of HBx in hepatocyte autophagy through SREBP2.

**Methods:**

The study employed TCGA and GEO databases to investigate the expression of SREBF2 and autophagy‐related proteins in liver cancer. Various experimental techniques, including dual‐luciferase reporter assays, immunohistochemistry, Western blotting, immunofluorescence, GFP‐mRFP‐LC3 puncta analysis, transmission electron microscopy, and Fillipin III staining, were conducted on HBV‐associated liver cancer tissues, HBV transgenic mice, and several liver cancer cell lines to assess the levels of HBx, SREBP2, autophagy, and cholesterol, respectively, as well as to explore potential associations between these factors.

**Results:**

Bioinformatics analysis suggested up‐regulation of SREBP2 and autophagy‐associated genes in HBV‐associated liver cancer. Elevated levels of cholesterol, SREBP2, and autophagy flux were detected in HBV‐associated liver cancer tissues as compared to adjacent tissues. HBV transgenic mice had higher cholesterol, SREBP2, and autophagy levels than wild‐type mice. HBx activated the SREBP2 promoter to enhance its transcription and nuclear translocation. HBx knockdown down‐regulated SREBP2 expression and nuclear translocation levels in HepG2.2.15‐siHBx cells. HepG2.2.15 and HepG2‐HBx showed more autolysosomes than HepG2 cells; furthermore, HepG2.2.15‐siHBx cells had fewer autolysosomes than HepG2.2.15 cells.

**Conclusions:**

This research highlights that HBx upregulates SREBP2 and increases autophagic flux, accompanied by changes in cholesterol metabolism, which offers an additional theoretical foundation to elucidate that chronic HBV infection causes abnormal lipid metabolism and induces tumorigenesis.

## Graphical abstract

This study reveals that Hepatitis B virus X protein can up‐regulate SREBP2 expression, augment cholesterol synthesis, and enhance autophagy flux, providing a new theoretical basis for the contribution of abnormal lipid metabolism caused by chronic HBV infection to the inflammatory–carcinoma transformation.

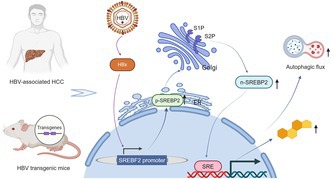

**Abbreviations**
ARBB1: arrestin beta 1
ATG: AuTophaGy
DAPI: 4′,6‐diamidino‐2‐phenylindole
ERS: endoplasmic reticulum stress
FASN: fatty acid synthase
FXR: farnesoid X receptor
GEO: Gene Expression Omnibus
HBV: Hepatitis B virus
HBx: Hepatitis B virus X protein
HCC: hepatocellular carcinoma
HMGCR: hydroxy‐3‐methylglutaryl‐CoA reductase
HMGCS1: 3‐hydroxy‐3‐methylglutaryl‐CoA synthase 1
LDLR: low‐density lipoprotein receptor
LXR: liver X receptor
MAP1LC3B/LC3B: microtubule‐associated protein 1 light chain 3 beta
MVK: mevalonate kinase
NASH: nonalcoholic steatohepatitis
NLRP3: NLR family pyrin domain containing 3
n‐SREBP2: nuclear SREBP2
PPAR: peroxisome proliferator‐activated receptor
p‐SREBP2: precursor SREBP2
SCAP: SREBP cleavage‐activating protein
SREBF2: sterol regulatory element binding transcription factor 2
SREBP2: sterol regulatory element binding protein 2
TCGA: The Cancer Genome Atlas

## 1 Introduction

Hepatitis B virus (HBV) infection is one of the significant causes of the initiation and progression of primary hepatocellular carcinoma (HCC), and approximately 50% of HCC is associated with chronic HBV infection [1]. Hepatitis B virus X (HBx) protein, a multifunctional regulatory protein encoded by the HBV X gene that can facilitate gene transcription and modulate cell growth, apoptosis, and DNA repair, is the most important viral protein in the onset and development of HCC [2, 3]. HBx acts as an autophagy receptor‐like molecule in the liver tissues of patients with chronic HBV infection and in cell lines transfected with HBV or HBx [4], suggesting HBx‐mediated autophagy as one of the pathophysiological mechanisms involved in chronic inflammation and tumorigenesis [5]. The formation of autophagosomes is evoked upon overexpression of the HBV genome or HBx protein [6, 7]. The specific mechanism by which HBx regulates autophagy is not yet fully understood. Increasing evidence suggests a dynamic role of autophagy in the genesis and development of HCC [8, 9]. During the malignant transformation of liver cells, autophagy exerts a promoting role in the development, metastasis, and drug resistance of HCC [10, 11, 12].

HBV infection is accompanied by significant abnormal lipid metabolism, and the initiation and development of liver cancer are highly dependent on energy metabolism processes, including lipogenesis and transformation [13]. A meta‐analysis has indicated that simultaneous liver fat deposition is associated with a high incidence of HCC [14]. The liver cholesterol levels were observed to be time‐dependently elevated in HBx transgenic mice [15]. Teng et al. reported that lipid metabolism‐associated genes were remarkably activated in the mouse model of HCC induced by transgenic expression of HBx and further validated in human HBV‐associated HCC, that the expression of these genes could alter the impact of HBx on lipid biosynthesis and restrain HBx‐induced proliferation in vitro [16]. It should not be ignored that abnormal lipid metabolism disrupts the endoplasmic reticulum and Golgi homeostasis, which further affects autophagy [17].

ChIP‐seq experiments verified that HBx could be enriched in autophagy‐regulatory genes, including SREBP2, Beclin‐1, and members of the AuTophaGy‐related (ATG) gene family [18]. SREBP‐2, encoded by sterol regulatory element binding transcription factor 2 (SREBF2), is one of the sterol regulatory element‐binding proteins (SREBPs) that primarily modulate liver cholesterol metabolism [19]. SREBP2 exists in the endoplasmic reticulum as precursor SREBP2 (p‐SREBP2) before shear activation, and when activated and sheared, it forms nuclear SREBP2 (n‐SREBP2), which enters the nucleus and participates in the regulation of cellular functions. Multiple studies have illustrated that the SREBP metabolic pathway is strongly linked to the genesis and development of liver cancer. Meanwhile, a study has also unveiled a regulatory effect of SREBP2 on autophagy genes during the activation process [20]. Whether HBx regulates hepatocyte autophagy through SREBP2, as well as its roles in liver diseases, particularly liver cancer, remains to be clarified. Therefore, this research seeks to clarify the regulatory role of HBx in hepatocyte autophagy through SREBP2. The experiments were conducted in three dimensions: human, animal, and cell.

## 2 Materials and Methods

### 2.1 Data Acquisition

An online analysis website, Xiantao Academic (https://www.xiantaozi.com/), contained RNA‐seq data from the TCGA database. TCGA Liver Cancer (LIHC) gene expression RNAseq and phenotype data were acquired from the UCSC Xena website (https://xenabrowser.net) to analyze and compare the expression of SREBF2 after matching the cancerous tissues with the adjacent tissues. Meanwhile, we screened and analyzed the difference in SREBF2 expression between hepatitis B‐related and other etiology‐related liver cancer tissues. Through the Assistant for Clinical Bioinformatics (https://www.aclbi.com/static/index.html#/geo), this study analyzed GEO datasets GSE121248 and GSE83148 for the comparison of SREBF2 expression. These two datasets from the GEO database (https://www.ncbi.nlm.nih.gov/geo/) were downloaded in MINIML format. GSE121248 data encompassed 37 adjacent tissues and 70 hepatitis B‐related cancerous tissues, and GSE83148 comprised 6 healthy and 122 HBV‐infected liver tissue samples.

### 2.2 Human Liver Samples

Liver cancer and adjacent pathological tissues were harvested from patients with concurrent HBV infection in our hospital from 2020 to 2023. Inclusion criteria: 50 paraffin‐embedded cancerous and adjacent tissue samples from HCC patients with chronic HBV infection preserved in the Department of Pathology and 20 frozen cancer and adjacent tissue samples preserved in the laboratory; Exclusion criteria: Concurrent infection with other hepatitis viruses (such as HCV), non‐viral hepatitis (such as alcoholic and non‐alcoholic steatohepatitis, drug‐induced hepatitis), acute hepatitis B, autoimmune hepatitis, primary biliary cholangitis, cholangiocarcinoma, metastatic liver cancer, etc. All procedures were granted by the Ethics Committee of our hospital (Ethic No: 2021KJCX032).

### 2.3 Animal Experimental Design

The mouse experiment was conducted by the hospital's ethical system. The experimental animals used in the present study: SPF adult wild‐type balb/c mice (16‐week‐old, weighing 27 ± 2 g, half male and half female) were provided by Shanghai SLRC Experimental Animal Co. Ltd. (Shanghai, China); Transgenic balb/c mice (16‐week‐old, 27 ± 2 g, half male and half female) harboring serotype ayw 1.3 genotype D HBV whole genome sequence were procured from Guangzhou Huateng Biopharmaceutical Technology Co. Ltd. (Guangdong, China). Animals were housed in a barrier environment with sufficient light under a light–dark cycle of 12 h. The temperature in the housing room was maintained at 23°C ± 3°C, and the humidity was 50%–60%. The animals were fed with a high‐pressure and irradiated feed with free access to food and water. A 1.5% ketamine solution was adopted for anesthesia before mouse euthanasia. All procedures were approved by the Laboratory Animal Ethics Committee of Fujian Medical University (Ethics Number: 2023‐Y‐0956).

### 2.4 Cell Culture and Plasmids

The human HCC cell lines (HepG2, Huh7, and HepG2.2.15) were stored in our laboratory. They were cultured in high‐glucose DMEM (Hyclone) replenished with 10% FBS and grown at 37°C in a 5% CO_2_ environment.

The plasmid expressing HBx, pcDNA3.1‐HBx (ayw) (Supporting Information S1: Doc S1.2), and the pcDNA3.1 vector were stored in our laboratory. siRNAs targeting HBx and SREBP2 were constructed by RiboBio (Supporting Information S1: Doc S1.1). The DNA of SREBP2 overexpression was constructed on the vector CMV‐MCS‐SV40‐Neomycin based on the transcript sequence of NM_004599 by Genechem (Supporting Information S1: Doc S1.3). The SREBP2 dual‐luciferase reporter gene plasmid was constructed on the MCS‐firefly_Luciferase‐PolyA‐Tk‐Renilla_Luciferase‐PolyA vector by Genechem (Supporting Information S1: Doc S1.4). Lipofectamine 3000 Reagent (L3000‐015, Thermo Fisher Scientific) was utilized for plasmid transfection. DNA synthesis rate assessment was conducted utilizing BeyoClick EdU‐647 Imaging Kits (C0081S, Beyotime), and experiments were finished with the method provided by the manufacturer. After overexpressing HBx in cells and knocking down SREBP2 according to the experimental requirements, perform the CCK‐8 (BS350B, Biosharp) cell proliferation assay as per the instruction manual, and measure the absorbance at 450 nm. The autophagy flux detection of HepG2 cells transiently transfected with HBx and Hepg2.2.15 was completed by intervention with rapamycin (HY‐10219, MedChemExpress) and bafilomycin A1 (HY‐100558, MedChemExpress), respectively.

### 2.5 Western Blotting

RIPA lysis buffer (Beyotime Biotechnology, Shanghai, China) was utilized for cell and liver tissue lysis. Following quantitative determination of protein concentration using a BCA assay kit (Beyotime, Shanghai, China), protein was separated on 7.5%, 12.5%, and 10% gels with sodium dodecyl sulfate (SDS) and transferred to a nitrocellulose membrane. Primary antibodies to HBx (1:1000, ab39716, Abcam), SREBP2 (1:1000, 10,007,663, Cayman), LC3B (1:1000, L7543, Sigma), P62 (1:1000, 88588S, Cell Cignaling Technology), Beclin1 (1:1000, 11,306‐1‐AP, Proteintech), and GAPDH (1:1000, 2118S, Cell Cignaling Technology) were added for overnight incubation at 4°C. The membrane was incubated for 1 h with secondary antibody, goat anti‐rabbit or mouse (1:5000, ZB‐2301 or ZB‐2305; ZSGB‐BIO) and rinsed with TBST three times (5 min) on the next day. Signals were detected with the ECL chemiluminescence system and interpreted using ImageJ Lab software.

### 2.6 Quantitative PCR

Cells were fragmented using Trizol, from which RNA was isolated with chloroform and then precipitated using isopropanol. The Revert Aid First Strand cDNA Synthesis Kit (K1622, Thermo, USA) was utilized for reverse transcription to synthesize cDNA. q‐RT‐PCR was conducted with the application of Fast Start Universal SYBR Green Master (ROX) (39,088,900, Roche, Germany). The fold changes in the expression of HBx and SREBF2 relative to β‐actin were calculated using the 2‐delta method. HBx primers: 5′‐ATGCAAGCTTATGGCTGCTAGGCTGTACTG‐3′ and 5′‐TGAGAATTCTTAGGCAGAGTGAAAAAGTTG‐3′; SREBF2 primers: 5′‐CCTGGGAGACATCGACGAGAT‐3′ and 5′‐TGAATGACCGTTGCACTGAAG‐3′; β‐actin primers: 5′‐CACAGACCTCGCCTTTGCC‐3′ and 5′‐TGACCCATGCCCACCATCAC‐3′.

### 2.7 Immunohistochemistry and Immunofluorescence

After antigen retrieval in EDTA/citrate, the paraffin sections were blocked with goat serum (ZLI‐9021, ZSGB‐BIO) and incubated overnight with diluted primary antibodies against SREBP2 (1:100, sc‐271,616, Santa Cruz), LC3B (1:50, 3868S, Cell Signaling Technology), P62 (1:100, 88588S, Cell Signaling Technology), and Beclin‐1 (1:800, ab62557, Abcam) at 37°C, followed by incubation with secondary antibodies (rabbit secondary antibody, PV‐9001, ZSGB‐BIO; mouse secondary antibody, PV‐9002, ZSGB‐BIO) the next day. The target protein was detected by staining with DAB dye (ZLI9018, ZSGB‐BIO), and the slices were counterstained with hematoxylin (ZLI9040, ZSGB‐BIO). After rinsing, dehydration, and hyalinization, the slices were sealed with resin and photographed under the microscope.

Tissue and cell immunofluorescence staining: cells were subjected to 20‐min fixation in 4% paraformaldehyde and then reacted with 0.5% Triton X‐100 (ST797, Beyotime) to increase membrane permeability. Primary antibody against SREBP2 (1:50, sc‐271,616, Santa Cruz) and corresponding secondary antibody (AlexaFluor594‐labeled goat anti‐mouse 1:1000, A‐11032, Thermo Fisher Scientific) were employed to detect target proteins. The nuclei were dyed with DAPI (1:10,000, D1306, Thermo Fisher Scientific) for 5 min and visualized and photographed under a confocal microscope (Leica, TSC SP8, Germany) with the use of an oil immersion lens.

### 2.8 Dual‐Luciferase Reporter Activity Assay

HepG2 and Huh7 cells were placed in a 24‐well plate and transfected with the SREBP2 promoter plasmid with pRL‐TK as the internal control. 48 h later, the collected cell lysate was subjected to dual‐luciferase activity detection as per the manufacturer's instructions (MCE, USA). With Renilla luciferase as the internal control, the ratio of the luminescence produced by firefly luciferase to the luminescence produced by Renilla luciferase was generated. The activation level of target reporter genes was compared among different samples based on the obtained ratio. Furthermore, Genechem Co. Ltd. was commissioned to construct different fragment truncations. The dual‐uciferase assay was then performed to identify the regulatory sites bound by HBx.

### 2.9 Filipin III Staining

Frozen tissue slices and cell crawlers were fixed with 4% paraformaldehyde for 1 h post two PBS rinses. Following three PBS rinses, the slices were soaked in glycine (1.5 mg/mL) for 20 min. An incubation with pre‐made Filipin III solution (SAE0087, Sigma, USA) was performed at 4°C overnight (in the dark) after PBS washes. After two PBS rinses, observation was achieved under an inverted ultraviolet‐excited fluorescence microscope (in the dark). Alternatively, PBS can be discarded before photographing after the addition of an anti‐quenching agent.

### 2.10 Lipid Quantification

The free cholesterol was determined by an enzymic method (E1016, Applygen). Animal tissues were first sliced and weighed, and then rinsed twice with PBS to remove residual blood. Every 1 mg of tissue was fragmented with the addition of 10 μL lysis buffer using an electric high‐speed homogenizer or a manual glass homogenizer. Cells were digested, centrifuged, and collected for cell counting. Add 0.1 mL of lysate to each 1 × 10^6^ cells, add 0.1 mL of lysate, and shake to mix. The tissues or cells were allowed to stand for 10 min, and then some supernatant was transferred to a 1.5 mL centrifuge tube, heated at 70°C for 10 min, and centrifuged at ambient temperature at 2000 g for 5 min. The harvested supernatant was added with a mixture of R1 and R2 (4:1) reagents to measure the OD value. The quantitative detection was realized through the cholesterol standard curve. Finally, cholesterol content was corrected for protein concentration.

### 2.11 mRFP‐GFP‐LC3B Puncta Assay

After cell seeding, cells were infected with mRFP‐GFP‐LC3B adenovirus for 48 h to express fluorescent LC3B. Before fluorescence detection, cells were stained with Hoechst 33342 live cell staining solution (Beyotime, C1028) for 10 min, rinsed 2 times in PBS, and supplemented with fresh culture medium. LC3B puncta were observed in cells using confocal immunofluorescence microscopy and statistically analyzed. The acidic environment inside the lysosome leads to the quenching of GFP fluorescence signals, and red fluorescent proteins have good tolerance to acidic environments. Cells stained with both RFP and GFP were defined as positive for autophagosomes, while RFP‐stained cells were defined as positive for autolysosomes.

### 2.12 Transmission Electron Microscopy (TEM)

Firstly, 1 × 1 × 1 mm^3^ of sectioned liver tissues or 1 × 10^6^ cells collected by digestion and centrifugation were immersed in 3% glutaraldehyde, 1.5% paraformaldehyde‐0.1 M PBS (pH 7.2) at 4°C for fixation. The tissues were rinsed in 0.1 M PBS. The tissues were reacted in 1% osmic acid‐1.5% potassium ferricyanide for 2 h, followed by fixation; the tissues were again rinsed with 0.1 M PBS. The tissues were gradually dehydrated in 50% ethanol for 10 min, 70% ethanol for 10 min, 70% ethanol‐saturated uranium acetate dye solution at 4°C overnight, 90% ethanol for 10 min, 90% ethanol‐acetone for 10 min, 90% acetone for 10 min, and anhydrous acetone 10 min; The tissues were completely immersed in anhydrous acetone + epoxy resin 618 (3:1) for 1 h, anhydrous acetone + epoxy resin 618 (1:1) for 1 h, and anhydrous acetone + epoxy resin 618 (1:3) for 1 h; embedding and polymerization were achieved using epoxy resin 618 at 35°C for 24 h, at 45°C for 12 h, and at 60°C for 2 days; the tissues were trimmed, sliced into semi‐thin sections, and positioned; then, 90–100 nm ultra‐thin sections were obtained with Leica UC‐7 ultra‐thin slicer; the sections were dyed by uranium acetate and lead citrate for 5–10 min successively and rinsed in distilled water, followed by observation and photography under FEI TECNA1 transmission electron microscope. To conduct accurate TEM quantification under our experimental conditions, we performed a quantitative comparative analysis on each TEM from 10 randomly selected fields of view based on the ultrastructures of autophagosomes, lysosomes, and autolysosomes.

### 2.13 Statistical Methods

Data were presented in the form of mean ± standard error (mean ± SD). One‐way analysis of variance (ANOVA) was adopted for analyzing data with homogeneous variance, followed by post hoc Tukey multiple comparison tests; data with non‐homogeneous variance were examined by a non‐parametric test (Kruskal‐Wallis rank sum) with Dunn's method for post hoc analysis. All data were statistically processed by GraphPad Prism V.8.0 (GraphPad Prism Software, San Diego, California, USA). Statistical significance was interpreted as *p* < 0.05.

## 3 Results

### 3.1 Elevated Expression of SREBF2 and Autophagy Genes in HBV‐Associated Liver Cancer

**FIGURE 1:**
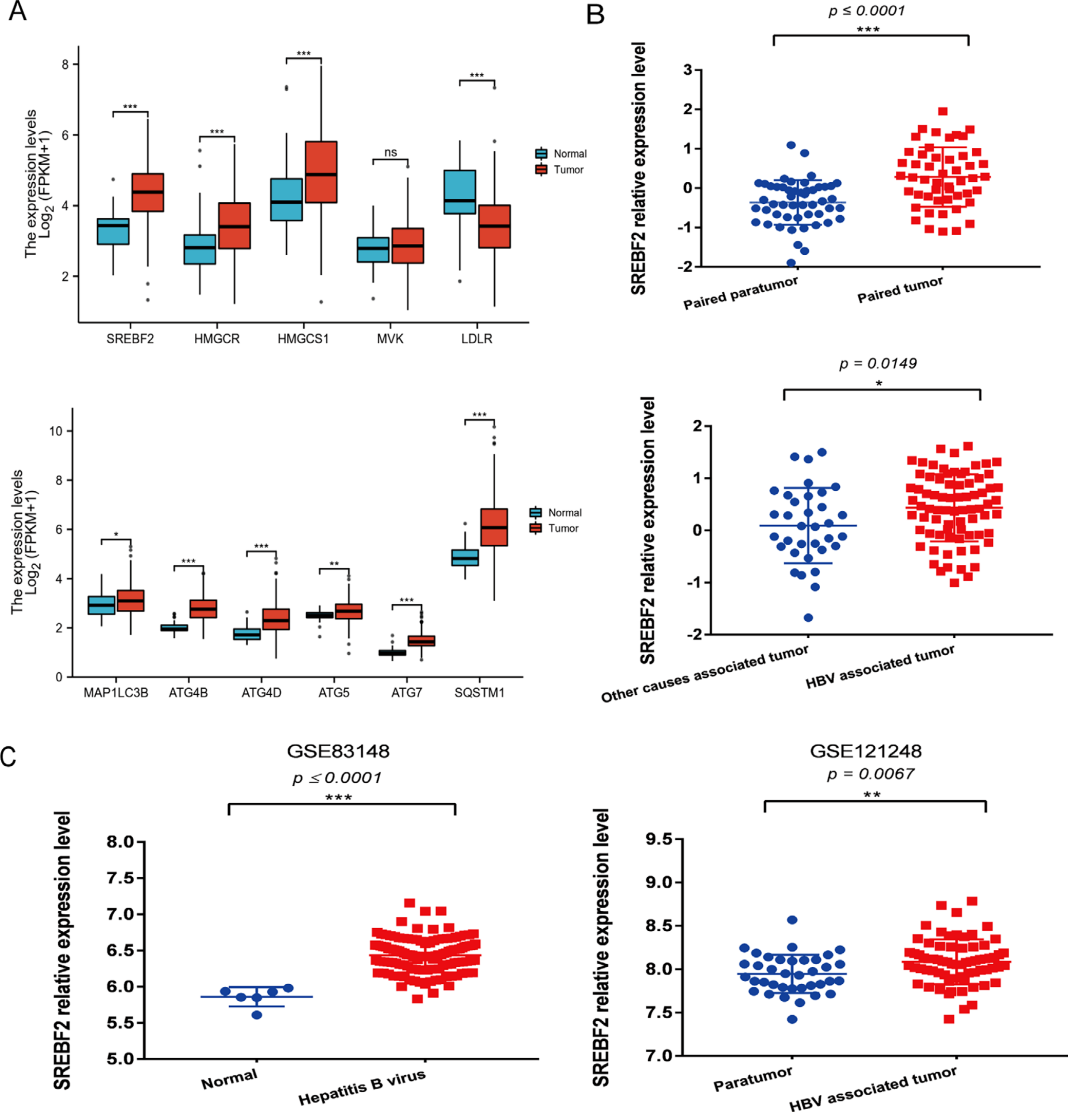
The expression of SREBF2 in HBV‐associated liver cancer based on TCGA and GEO databases. (A) Expression of cholesterol metabolism‐related genes and autophagy‐related genes at transcriptional levels in the TCGA database. (B) Using TCGA pan‐cancer datasets, SREBF2 expression was further compared between hepatitis B‐related liver cancer tissues and non‐hepatitis B‐related liver cancer tissues. (C) According to GEO datasets GSE83148 and GSE121248, SREBF2 expression was compared between cancerous and normal tissues as well as between hepatitis B‐related hepatitis tissues and normal liver tissues. *p < 0.05, **p < 0.01, ***p < 0.001.

The results of hepatitis B‐related liver cancer data analysis suggested higher expression of SREBF2 and autophagy indicators in cancerous tissues than in adjacent tissues. From the TCGA database, we found that in 424 unmatched samples (374 liver cancer tissue samples, 50 adjacent normal tissue samples) (Figure 1A), transcription levels of SREBF2 and some downstream cholesterol metabolism‐related genes (HMGCR, HMGCS1, LDLR, etc.) were mostly higher in cancerous tissues versus adjacent tissues; the transcription levels of selected autophagy‐associated genes were also higher in cancerous tissues than in adjacent tissues. SREBF2 was again compared after matching the cancerous tissues with the adjacent tissues, which still indicated higher expression in the cancerous tissues than in the adjacent tissues. The expression of SREBF2 in hepatitis B‐related liver cancerous tissues was further higher than that in non‐hepatitis B‐related cancerous tissues (Figure 1B). SREBF2 expression in GEO datasets GSE83148 and GSE121248 was analyzed (Figure 1B,C). GSE83148 contained 6 healthy and 122 HBV‐infected liver tissue samples, the results of which showed that SREBF2 expression was significantly higher in hepatitis B tissues than in normal liver tissues. GSE121248 data involved 37 adjacent normal tissue samples and 70 hepatitis B‐related cancerous tissue samples. Their comparison results indicated significantly higher SREBF2 expression in the cancerous tissues relative to normal tissues.

### 3.2 Elevated SREBP2 Expression and Nuclear Translocation of SREBP2 as well as Augmented Autophagy in Hepatitis B‐Associated Liver Cancer Tissues

**FIGURE 2:**
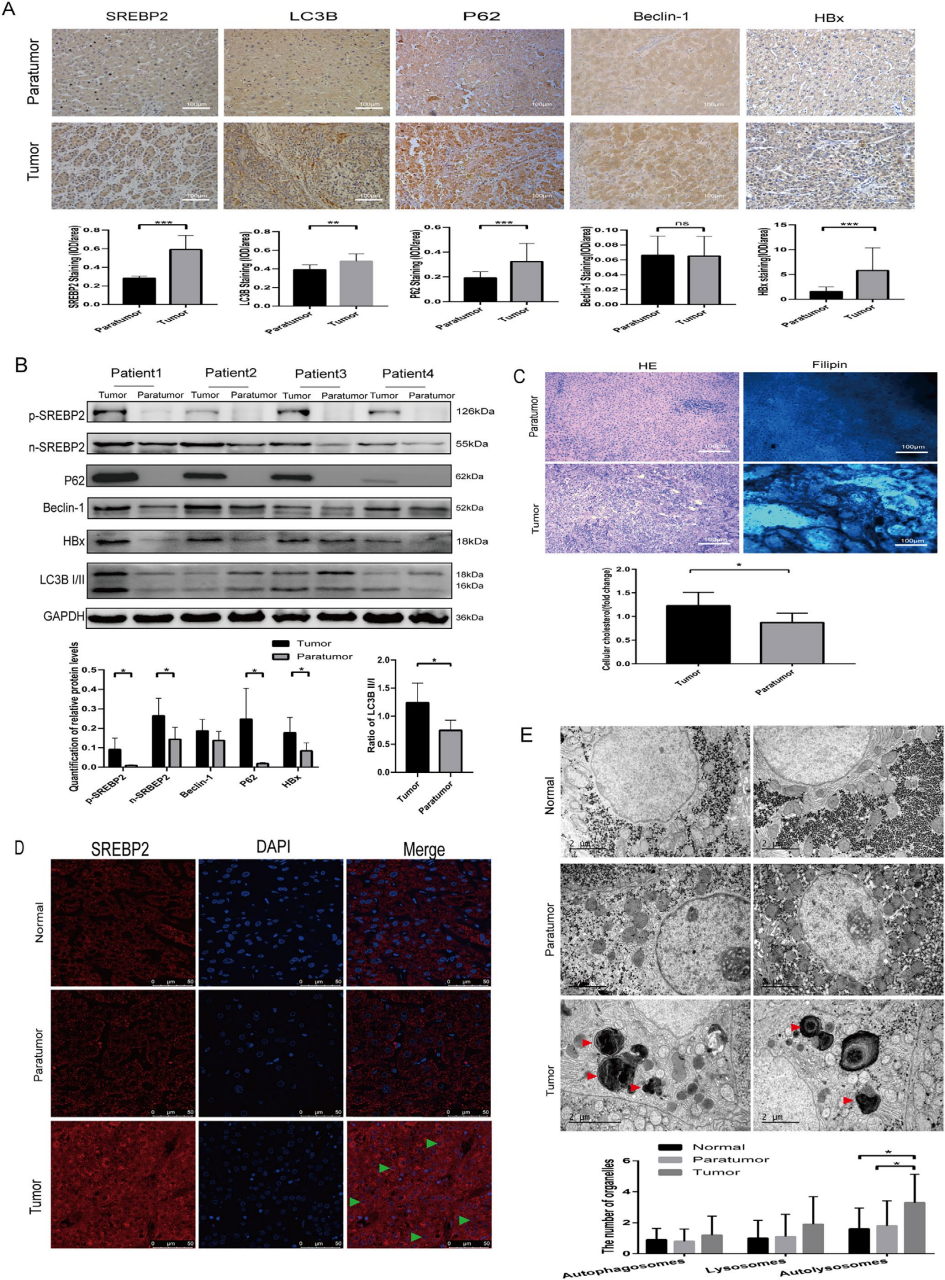
Up‐regulation of SREBP2 and cholesterol levels and increased autophagy in HBV‐associated liver cancer tissues. (A) Immunohistochemical detection of LC3B, P62, Beclin1, SREBP2 and HBx expression in liver cancer and corresponding adjacent tissues of patients, with microscopic fields of view (200×); the average cumulative positive rate (IOD/area) of images were calculated by Image J software and analyzed by paired *t*‐tests; the values were expressed in mean ± SD. (B) Detection of SREBP2 expression and autophagy‐associated proteins in liver cancer and adjacent pathological tissues of four patients with concurrent HBV infection. (C) Detection of cholesterol levels in liver cancer and adjacent pathological tissues of patients with concurrent HBV infection using Filipin III staining and ELISA. (D) Immunofluorescence observation of SREBP2 nuclear translocation in liver cancer, adjacent tissues, and normal liver tissues of patients with HBV infection (green arrows) (confocal 63x oil immersion lens). (E) More autolysosomes (red arrows) were observed in liver cancer tissues than in adjacent tissues through electron microscopy.*p < 0.05, **p < 0.01, ***p < 0.001.

Expression of SREBP2 and autophagy‐associated proteins in clinical samples was detected through immunohistochemical techniques (Figure 2A). It was found that SREBP2 and autophagy‐associated proteins LC3B and P62 were expressed at higher levels in liver cancer tissues than in adjacent tissues. Changes in the levels of p‐SREBP2, n‐SREBP2, HBx, and autophagy markers in the pathological tissues of HBV‐associated HCC patients were assessed by Western blotting (Figure 2B). It was suggested that the full‐length and activation level of SREBP2 and the LC3B I/II conversion rate increased in cancerous tissues in contrast to adjacent tissues. Increased nuclear translocation of SREBP2 was observed in liver cancer tissues in patients suffering from hepatitis B‐associated liver cancer. The results of Filipin III staining and ELISA revealed an elevation in the cholesterol level in cancerous tissues in comparison with adjacent tissues (Figure 2C). Immunofluorescence was then carried out to visualize the nuclear translocation of SREBP2 in liver cancer, adjacent normal, and normal tissues of patients with HBV infection (Figure 2D). The autophagy levels of hepatitis B‐relevant liver cancer tissue, adjacent tissues, and normal liver tissues were observed under the electron microscope. A significant increase was noted in the quantity of autolysosomes in liver cancer tissue as compared to adjacent tissues and normal liver tissues (Figure 2E). Therefore, SREBP2 was up‐regulated in cancer tissues, accompanied by increased translocation into the nucleus and enhanced autophagy.

### 3.3 Increased SREBP2 Expression and Autophagy Levels in HBV Transgenic Mice

**FIGURE 3:**
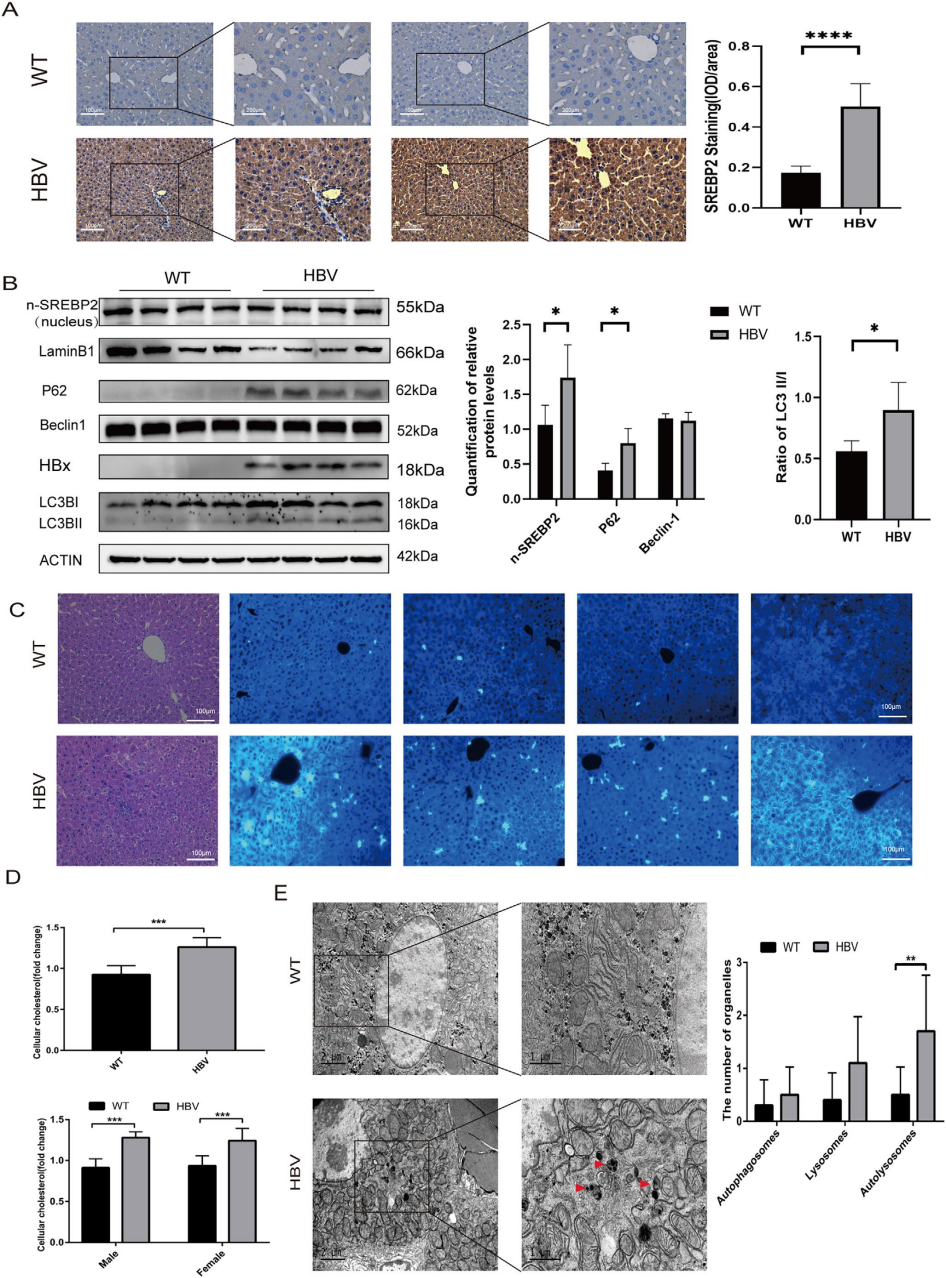
Up‐regulation of SREBP2 and cholesterol levels and increased autophagy in HBV transgenic mice. (A) Immunohistochemical analysis of SREBP2 expression in liver tissues of wild‐type mice and HBV transgenic mice. (B) Western blotting was used to detect SREBP2 activation and nuclear translocation levels and autophagy‐related proteins in HBV transgenic mice and wild‐type mice. (C) Filipin III staining was used to detect cholesterol levels in liver tissues of HBV transgenic mice and wild‐type mice. (D) ELISA was used to detect cholesterol levels in the liver tissues of HBV transgenic mice and wild‐type mice. (E) Autophagosomes and autophagolysosomes (red arrows) were observed through transmission electron microscopy in hepatocytes of HBV transgenic mice and wild‐type mice.*p < 0.05, **p < 0.01, ***p < 0.001.

Immunohistochemical tests showed higher SREBP2 expression in HBV transgenic mice in comparison with wild‐type mice (Figure 3A). Western blotting data exhibited that the nuclear SREBP2, LC3BII, and P62 levels were higher in HBV transgenic mice than in wild‐type mice (Figure 3B). Additionally, higher cholesterol levels detected by Filipin III staining (Figure 3C) and an elevation in cholesterol expression detected by ELISA were observed in HBV transgenic mice (Figure 3D) in comparison with the wild‐type mice. Through TEM observation, increased autolysosomes were visible in HBV transgenic mice (Figure 3E), which was consistent with the aforementioned results from human tissues. After HBV infection, SREBP2 expression was up‐regulated, and the autophagy level was augmented.

### 3.4 HBx Up‐Regulates SREBP2 Expression in Hepatocytes and Facilitates Its Nuclear Translocation

**FIGURE 4:**
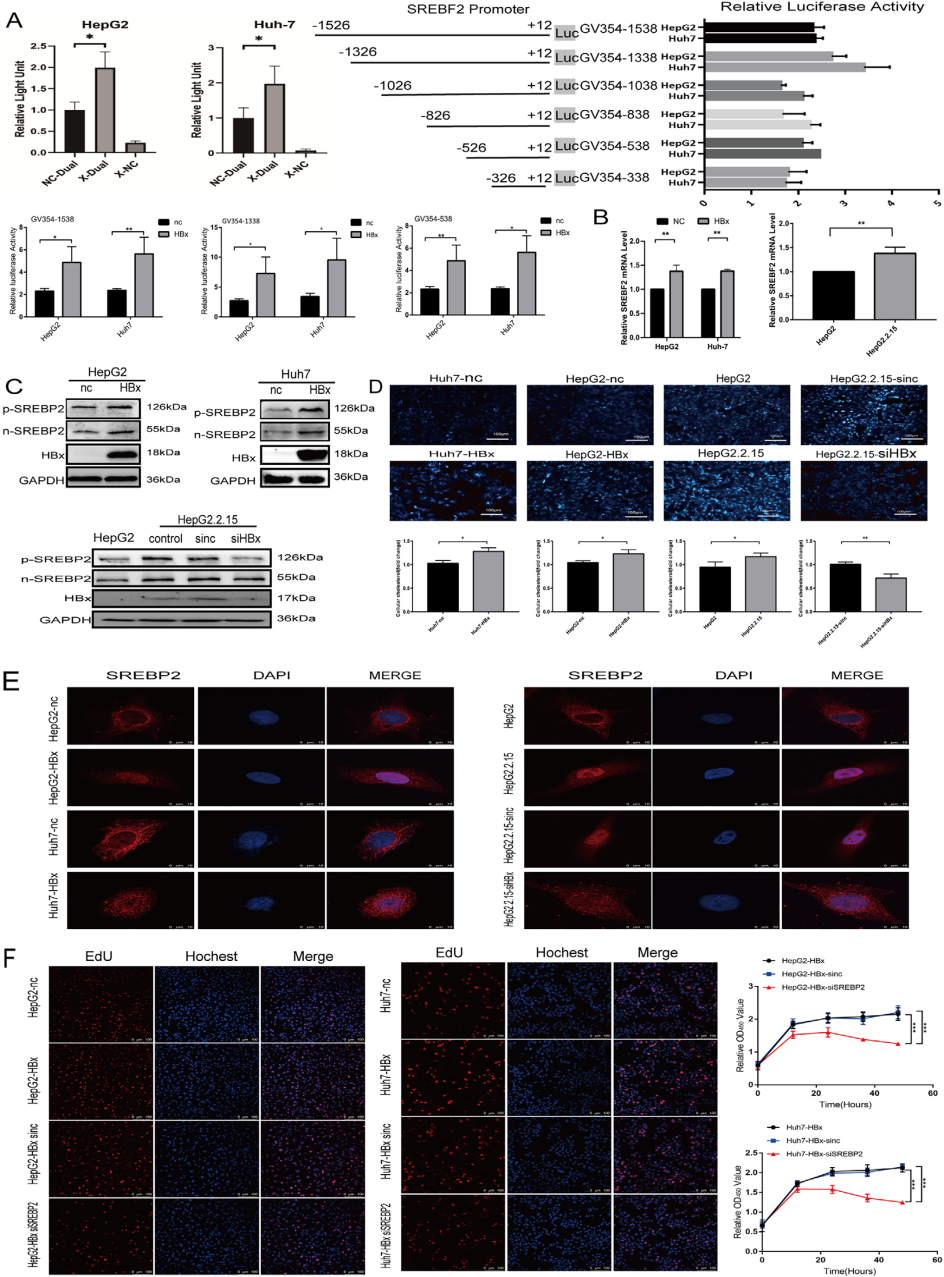
The expression and nucleation status of SREBP2 in cells. (A) Dual‐luciferase reporter gene assay to assess the direct activation of SREBP2 promoter by HBx; And construct different fragment truncations for the dual‐luciferase assay to identify the possible regulatory sites of HBx. (B) PCR detection of SREBF2 expression in HepG2 and Huh‐7 cells overexpressing HBx and HepG2.2.15 cells; (C) HepG2 and Huh‐7 cells overexpressing HBx, HepG2.2.15, and HBx‐silenced HepG2.2.15 cells were constructed, and the expression and activation of SREBP2 were detected using Western blotting technique; (D) FilipinIII and ELISA detection results of HepG2, Huh‐7, and HepG2.2.15 cells; (E) Immunofluorescence detection of the SREBP2 activated fragments and nucleus co‐localization; (F) Observe the effect of SREBP2 on cell proliferation through the EDU proliferation assay.*p < 0.05, **p < 0.01, ***p < 0.001.

HepG2 and Huh‐7 cell lines were co‐transfected with the HBx plasmid and dual‐luciferase plasmid containing the SREBP2 promoter. The data suggested that HBx activated the SREBP2 promoter sequence (Figure 4A). We further identified the core region of the SREBF2 promoter. SREBF2 plasmids of different lengths, including −1526/+12 (GV354–1538), −1326/+12 (GV354–1338), −1026/+12 (GV354–1038), −826/+12 (GV354–838), −526/+12 (GV354–538), and −326/+12 (GV354–338), were cloned and transiently transfected into HepG2 (or Huh7) cells to measure promoter activity. Luciferase reporter gene assays showed that GV354–1538 and GV354–1338 exhibited the highest luciferase activities (Figure 4A), indicating that the −1326/+12 region might be the core promoter region of SREBF2. Co‐transfection of an HBx overexpression plasmid in HepG2 and Huh7 cell lines, respectively, significantly increased the luciferase activity (Figure 4A).

Overexpression of HBx in HepG2 and Huh‐7 cell lines up‐regulated SREBP2 transcription and translation levels as well as its nuclear translocation level (Figure 4B,C). After knocking HBx down in HepG2.2.15 cells by transfection with siHBx, the expression of full‐length and activated fragment SREBP2 was down‐regulated (Figure 4C). The Filipin staining and ELISA results suggested an elevation in cholesterol levels in the HBx overexpression group (Figure 4D). Confocal immunofluorescence observation showed an increase in SREBP2 nuclear translocation upon HBx overexpression, and HepG2.2.15 cells exhibited more significant SREBP2 nuclear translocation than HepG2 cells. HBx knockdown resulted in a decrease in SREBP2 nuclear translocation in HepG2.2.15‐siHBx (Figure 4E). Both the EdU and CCK8 cell proliferation assays demonstrated that the proliferation of hepatocellular carcinoma cells with overexpressed HBx significantly decreased after SREBP2 was knocked down, suggesting that HBx may promote cell proliferation through SREBP2 (Figure 4F).

### 3.5 HBx Regulates Cell Autophagy Through SREBP2

**FIGURE 5:**
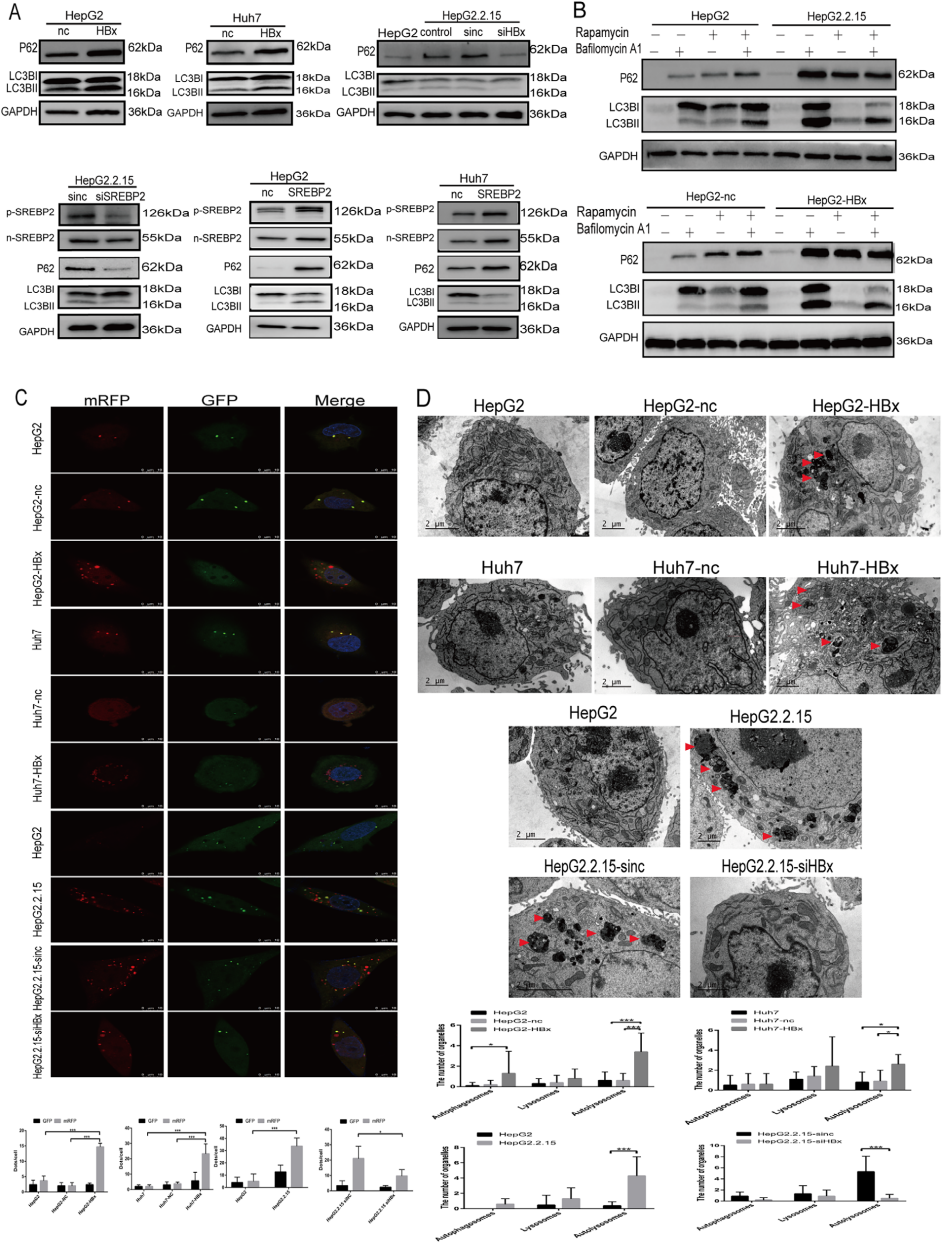
HBx regulates cell autophagy through SREBP2. (A) The expression of autophagy markers in HepG2‐HBx, Huh7‐HBx, HepG2.2.15, and HepG2.2.15‐siHBx cells was measured by Western blotting techniques, as well as the changes in autophagy after SREBP2 knockdown or overexpression. (B) After HepG2 and HepG2.2.15 cells were seeded for 24 h, rapamycin (working concentration 30 uM) and bafilomycin A1 (working concentration 10 nM) were administered for 24 h according to the autophagy flux detection method. Similarly, after HepG2 cells were transiently transfected with HBx for 24 h, they were also treated with rapamycin and bafilomycin A1 for 24 h. (C) The autophagy of HepG2‐HBx, Huh7‐HBx, HepG2.2.15, and HepG2.2.15‐siHBx cells was assessed through the Puncta assay. (D) Autolysosomes (red arrows) in HepG2‐HBx, Huh7‐HBx, HepG2.2.15, and HepG2.2.15‐siHBx cells were observed through transmission electron microscopy.*p < 0.05, **p < 0.01, ***p < 0.001.

Western blotting technique was employed to detect the alterations of autophagy markers in HepG2‐HBx, Huh7‐HBx, HepG2.2.15, and HepG2.2.15‐siHBx cells, as well as the changes in autophagy after SREBP2 overexpression or knockdown (Figure 5A). HBx overexpression was suggested to augment autophagy levels; either HBx or SREBP2 knockdown could inhibit autophagy. The autophagy markers in HepG2‐SREBP2 and Huh7‐SREBP2 cells were elevated in response to SREBP2 overexpression. These obtained results were consistent with in vivo experimental results, indicating that HBx could mediate SREBP2 to upregulate autophagy. To further distinguish whether HBx promotes early autophagy or influences the late stage of autophagy, we further observed the changes in autophagy flux by intervening with rapamycin and bafilomycin A1. When treated with rapamycin alone, the content of LC3BII was increased in both types of cells. The combined treatment with rapamycin and bafilomycin A1 further increased the content of LC3BII in both types of cells (Figure 5B). It can also be seen from the figure that in both types of cells treated with the autophagosome‐lysosome fusion inhibitor without rapamycin treatment, the content of LC3BII in HepG2.2.15 cells was significantly higher than that in HepG2 cells. Through the mRFP‐GFP‐LC3B puncta experiment, it was found that the number of autolysosomes was higher in HepG2 and Huh7 cells overexpressing HBx than in HepG2‐NC and Huh7‐NC, respectively. HepG2.2.15 cells had more autolysosomes than HepG2 cells, and HepG2.2.15‐siHBx cells had fewer autolysosomes than HepG2.2.15 cells (Figure 5C). The autolysosomes and lysosomes in HepG2‐HBx, Huh7‐HBx, and HepG2.2.15 cells, as well as the autophagy levels of HepG2.2.15‐siHBx cells after HBx knockdown, were observed by electron microscopy. The results showed an increased number of autolysosomes in HepG2‐HBx and Huh7‐HBx cells as compared to the control HepG2 and Huh7 cells. HepG2.2.15 cells had more autolysosomes than HepG2 cells, and HepG2.2.15‐siHBx cells had a reduced number of autolysosomes as compared to HepG2.2.15 cells (Figure 5D). Therefore, HBx overexpression affected cholesterol metabolism and increased autophagic flux by up‐regulating SREBP2.

## 4 Discussion

HBV infection represents a principal public health threat worldwide. From the perspective of the interaction mechanism between HBV and lipid metabolism, HBx performs a crucial role in HBV‐evoked hepatic steatosis [21]. HBx promotes de novo synthesis and in vitro uptake of intracellular lipids in cell and mouse models, increasing cellular lipid accumulation [22]; conversely, important regulatory pathways for lipid metabolism, such as PPAR, LXR, and FXR, can affect intracellular HBV replication at the transcriptional level [23, 24]. Lipidomics evidence suggests that the accumulation of cholesterol esters has a tumor‐promoting effect in the development of HCC [25]. The concept of regulating cholesterol metabolism for the treatment of metabolic‐related cancers has been clinically applied [26]. For instance, the anticancer properties of Statins have gained much attention, but their effectiveness remains controversial [27]. Unlike the ability of HBV to promote lipid synthesis and intake observed in in vitro experiments and animal experiments, some clinical studies and meta‐analyses have shown that the incidence of fatty liver diseases is relatively low in HBV‐infected individuals [28]. Both abnormal cholesterol metabolism and chronic HBV infection can expedite hepatocarcinogenesis and cancer progression, but their relationship is still not clarified [29].

In this study, information from TCGA and GEO databases suggested that the transcription levels of SREBP2 and its downstream key cholesterol regulators, including (HMGCR, HMGCS1, MVK, etc.) as well as the levels of several autophagy‐associated proteins, were up‐regulated in HBV‐associated cancer tissues. The Western blotting and immunohistochemical results of clinical liver cancer samples in our research were consistent with the bioinformatics data. Meanwhile, immunofluorescence data suggested an enhancement in SREBP2 nuclear translocation in cancerous tissues; Filipin III staining and ELISA results exhibited increased cholesterol content in liver cancer tissues versus adjacent tissues, suggesting a significant impact of SREBP2 on the modulation of cholesterol metabolism and autophagy in the occurrence and development of HBV‐associated liver cancer. Given the interaction as shown by the CHIP‐seq results [18], we further validated the interaction between HBx and SREBP2 through dual‐luciferase reporter gene experiments. HBV transgenic mice exhibited higher SREBP2 expression, cholesterol content, and autophagy levels versus wild‐type mice. Subsequent cell experimental data validated that HBx up‐regulated the SREBP2 protein levels in HepG2, Huh7, and HepG2.2.15 cell lines and that SREBP2 expression was down‐regulated when HBx expression was inhibited in HepG2.2.15 cells, which further substantiated the regulatory effect of HBx on SREBP2. SREBP2 affects the proliferation level of hepatocellular carcinoma cells, and inhibiting or enhancing SREBP2 could induce corresponding changes in autophagy levels in cells.

Autophagy is engaged in the initiation and progression of HBV‐associated liver cancer [5, 30], but the regulatory effects of HBx on autophagy at different stages of disease progression after chronic HBV infection remain controversial [31, 32]. Given the extensive development of autophagy‐regulatory drugs, clarifying the significance of autophagy in the initiation and development of HBV‐associated liver cancer can provide more evidence for the development of clinical therapeutic regimens. This study documented significant elevations in LC3B expression and the LC3BII/I ratio in human liver cancer tissues relative to adjacent tissues. In HepG2 and Huh7 cells transfected with the HBx gene and HepG2.2.15 cells stably transfected with HBV, it was found that HBx promoted LC3BI/II conversion. We further observed the changes in autophagy by jointly intervening in the cells with rapamycin and bafilomycin A1. The results indicated that the basal level of autophagy in cells stably infected with HBV was higher than that in cells not infected with HBV. When autophagy was induced in cells, the magnitude of the increase in autophagy flux in cells stably infected with HBV was higher than that in the control cells, and this enhancement of autophagy occurred at an early stage. In the cells that were treated with the autophagosome–lysosome fusion inhibitor but without rapamycin treatment, the content of LC3BII in HepG2‐HBx cells was significantly higher than that in HepG2 cells. This result indicates that the basal autophagy level of HepG2‐HBx cells is higher than that of HepG2 cells. When autophagy was induced in cells, the magnitude of the increase in autophagy flux in HepG2‐HBx cells was higher than that in the control cells, and this enhancement of autophagy occurred at an early stage. In order to further evaluate the autophagy flux, we performed puncta experiments with dual staining of LC3 and found that the number of autolysosomes was significantly higher in liver cancer cells transfected with HBx and HepG2.2.15 cells than in the empty vector group. After silencing HBx with siRNA, the promoting effect of HBx on autophagy was partially blocked. The electron microscopic observation also indicated that the numbers of autophagosomes and autolysosomes were increased in liver cancer cells transfected with HBx and HepG2.2.15 cells as compared to empty controls. Meanwhile, it was shown that autophagosomes and autolysosomes were remarkably elevated in HBV transgenic mice and human HBV‐associated liver cancer tissues versus wild‐type mice and adjacent cancer tissues, respectively. All of these supported the promoting autophagic effect of HBx. However, the immunohistochemistry results of human liver samples suggested that P62 also increased when LC3BII/I increased. This finding seems to be inconsistent with the increased autophagic flow supported by other experimental results, suggesting the existence of other pathways regulating P62. Multiple pieces of evidence support that HBx protein directly promotes the aggregation and up‐regulation of P62 and restrains liver cancer cell apoptosis by up‐regulating P62 expression [33, 34]. On the other hand, P62, as a key selective autophagy adapter, participates in selective autophagy. Recently, new evidence supports that P62 acts as a key signal transduction connector in the tumor microenvironment, surpassing its role in autophagy. P62 performs a complex environment‐dependent role in the metabolic reprogramming of tumor and stromal cell types, thereby shaping the tumor microenvironment to control tumor progression [35, 36]. Therefore, the up‐regulation of P62 may be influenced by multiple pathways. Wei et al. also reported that P62 and autophagy synergistically accelerated tumor growth [37]. In this regard, some scholars proposed that both autophagy and P62 must be suppressed in some tumors to successfully implement strategies to regulate autophagy against cancer [38].

The SREBP signaling pathway regulates cellular sterol homeostasis through a series of negative feedback mechanisms. Existing research has attained considerable understanding of the regulatory role of SREBP1 in lipid metabolism. Kim et al. confirmed elevations in SREBP‐1 and PPARγ expression in mice expressing HBx protein (HBx‐Tg mice) and HepG2 cells stably introduced with HBx [39], and HBx located in the nucleus has a stronger activation effect on SREBP‐1 [40]. Multiple clinical research results and metabolomics suggest that abnormal cholesterol metabolism, like abnormal triglyceride metabolism, affects the carcinogenic mechanism of HBV, but the regulatory role of SREBP2 in this process remains undefined. SREBP2 is a key modulator of cellular cholesterol biosynthesis [41]. Under physiological conditions, the regulation of cholesterol metabolism by SREBP2 is a dynamic equilibrium process [42]. Based on this premise, increased activity of SREBP2 indicates a low cholesterol level. Conversely, an increase in cellular cholesterol levels can lead to a decrease in SREBP2 activity [20]. For HBV‐infected individuals, although the cholesterol levels in hepatocytes are at normal levels, the sustained expression of HBx upon HBV replication leads to sustained abnormal activation of SREBP2, which in turn results in enhanced cholesterol metabolism and autophagy levels in hepatocytes. Our results in the clinicopathological sections also substantiated this point. It has been suggested that SREBP2 can directly or indirectly affect the occurrence and development of HCC [43]. In a FASN gene‐knockout hepatic neoplasm mouse model, HMGCR cholesterol synthesis and SREBP‐2 nuclear translocation are elevated [44]. The sustained abnormal activation of SREBP2 provides conditions for NLRP3 inflammasome activation [45], and its induced increase in cholesterol synthesis is lipotoxic, making it an important pathogenic factor for NASH [46] and FASN deficiency‐induced HCC^44^; on the other hand, after liver cells undergo malignant transformation into liver cancer cells, the autophagy induced by SREBP2 activation performs anti‐apoptotic and pro‐invasive roles in the cells [47]. In large‐scale epidemiological studies, the application of Statins is relevant to a reduced risk of HCC development, indicating that regulating the cholesterol biosynthesis pathway may be an effective method for the prevention of liver cancer [48]. How does the abnormal expression of SREBP2 regulate the changes in autophagy after HBV infection? Previous studies demonstrated that HBV can elicit endoplasmic reticulum stress (ERS) [49], while inhibiting SREBP2 nuclear translocation can alleviate ERS [50]. Thus, whether HBx‐mediated SREBP2 affects autophagy by triggering ERS remains to be further explored.

**FIGURE 6:**
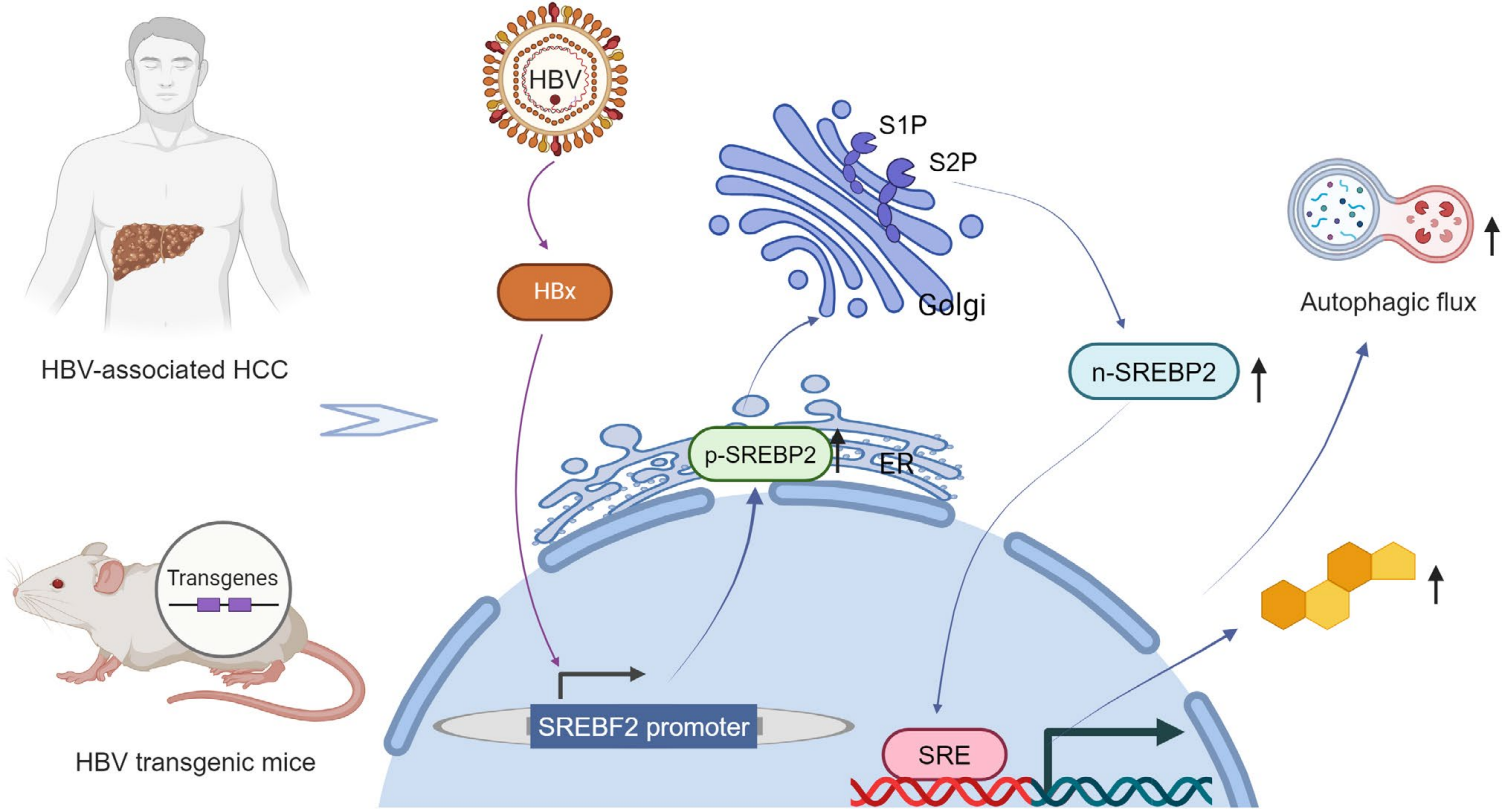
The proposed model shows the cross‐talk between lipid metabolism and HBV in HCC.

Conclusively, our experimental results offered new evidence for the involvement of lipid metabolism and HBV crosstalk in HCC (Figure 6), providing a new perspective for exploring therapeutic methods of HCC.

## Author Contributions

**Qiuyan Lin:** investigation (equal), writing – original draft (equal). **Yongxu Lin:** resources (equal), writing – original draft (equal). **Yongzhu Huang:** conceptualization (equal), resources (equal). **Mingrong Wang:** investigation (equal). **Xiaoxia Xie:** investigation (equal). **Weiqi Cai:** conceptualization (equal). **Qilan Guo:** data curation (equal). **Pingying Jiang:** data curation (equal). **Yuanlin Qi:** supervision (lead). **Dan Li:** supervision (equal), writing – review and editing (equal).

## Ethics Statement

All procedures were granted by the Ethics Committee of Fujian Medical University Union Hospital (approval number Medical Ethics: 2021KJCX032). All animal procedures were approved by the Laboratory Animal Ethics Committee of Fujian Medical University (approval number Ethics: 2023‐Y‐0956).

## Consent

The participants signed an informed written letter of consent.

## Conflicts of Interest

The authors declare no conflicts of interest.

## Supporting information


Data S1.

## Data Availability

The data that support the findings of this study are available from the corresponding author upon reasonable request.
