# Supplementary material for: Hepatitis B Virus X Protein Upregulates SREBP2 to Modulate Autophagy in Hepatocellular Carcinoma
Source: Cancer Med. 2025 Jun 6;14(11):e70916. doi: 10.1002/cam4.70916 (PMC12142432; doi:10.1002/cam4.70916)
Supplement: Supplementary file 1 — Data S1. [file CAM4-14-e70916-s001.doc]

**S1.1** siRNA

| Gene |  | Sequence(5’-3’) |
| --- | --- | --- |
| siHBx  siSREBP2 | Sense  Sense | AAGAGGACUCUUGGACUCUCA  GAGGCAGGCUUUGAAGACGAAGCUA |

**S1.2** pcDNA3.1-HBx(strain ayw)

ATGGCTGCTAGGCTGTGCTGCCAACTGGATCCTGCGCGGGACGTCCTTTGTTTACGTCCCGTCGGCGCTGAATCCTGCGGACGACCCTTCTCGGGGTCGCTTGGGACTCTCTCGTCCCCTTCTCCGTCTGCCGTTCCGACCGACCACGGGGCGCACCTCTCTTTACGCGGACTCCCCGTCTGTGCCTTCTCATCTGCCGGACCGTGTGCACTTCGCTTCACCTCTGCACGTCGCATGGAGACCACCGTGAACGCCCACCAAATATTGCCCAAGGTCTTACATAAGAGGACTCTTGGACTCTCAGCAATGTCAACGACCGACCTTGAGGCATACTTCAAAGACTGTTTGTTTAAAGACTGGGAGGAGTTGGGGGAGGAGATTAGGTTAAAGGTCTTTGTACTAGGAGGCTGTAGGCATAAATTGGTCTGCGCACCAGCACCATGCAACTTTTTCACCTCTGCCTAA

**S1.3** pcDNA3.1-SREBP2

Vector：CMV-MCS-SV40-Neomycin

Transcription number: NM_004599( Gene ID:6721)

NM_004599: CTTTCTGTGCGGCGCCCGGGCGCAACGCAAACATGGCGGCGGGGTGGCACCCGTCGGTGAGCGGGTGCCGGGCGGGGGTTGTCGGGTGTCATGGGCGGTGGCGACGGCACCGCCCCCGCGTCTCCCTGAGCGGGACGGCAGGGGGGGCTTCTGCGCTGAGCCGGGCGATGGACGACAGCGGCGAGCTGGGTGGTCTGGAGACCATGGAGACCCTCACGGAGCTGGGCGACGAGCTGACCCTGGGAGACATCGACGAGATGCTGCAATTTGTCAGTAATCAAGTGGGAGAGTTCCCTGACTTGTTTTCAGAACAGCTGTGTAGCTCCTTTCCTGGCAGTGGTGGTAGTGGTAGCAGCAGCGGCAGCAGTGGCAGCAGCAGCAGCAGCAGCAATGGCAGGGGCAGCAGCAGCGGAGCTGTGGACCCTTCAGTGCAACGGTCATTACCCAGGTCACATTACCTTCCTTCTCTCCCTCGGCGGCTCCCCACAGGCTCCAACTCTGCAAGTCAAGGTTTCTCCCACCTCAGTTCCCACCACACCCAGGGCAACTCTTATTCTTCAGCCCCGCCCCCAGCCCCAGCCTCAACCTCAAACTCAGCTGCAACAACAGACGGTAATGATCACGCCAACATTCAGCACCACTCCGCAGACGAGGATCATCCAGCAGCCTTTGATATACCAGAATGCAGCTACTAGCTTTCAAGTCCTTCAGCCTCAAGTCCAAAGCCTGGTGACATCCTCCCAGGTACAGCCGGTCACCATTCAGCAGCAGTGCAGACAGTACAGGCCCAGC GGG TGCTGACACAAACGGCCAATGGCACGCTGCAGACCCTTGCCCCGGCTACGGTGCAGACAGTTGCTGCGCCACAGGTGCAGCAGGTCCCGGTCCTGGTCCAGCCTCAGATCATCAAGACAGATTCCCTTGTTTTGSSACCACACTGAAGACAGATGGCAGCCCTGTTATGGCTGCGGTCCAGAACCCGGCCCTCACCGCCCTCACCACCCCCTATCCAGACGGCTGCCCTTCAAGTACCAACCCTGGTGGGCAGCAGTGGGACCATTCTGACCACAATGCCTGTAATGATGGGGCAAGAGAAAGTGCCCATTAAGCAGGTACCTGGGGAGTCAAGCAGCTTGAGCCCCCCCAAAGAAGGAGAAAGGCGGACAACCCATAATATCATTGAGAAACGATATCGCTCCTCATCAATGACAAAATCATCGAATTGAAAGACCTGGTCATGGGGACAGACGCCAAGATGCACAAGTCTGGCGTTCTGAGGAAGGCCATTGATTACATCAAATACTTGCAGCAGTGTCAATCATAAAACTGCGCCAGGAGAACATGGTGCTGAAGCTGGCAAATCAAAAGAACAAGCTTCTAAAGGGCATCGACCTAGGCAGTCTGGTGGACAATGAGGTGGACCTGAAGATCGAGGACTTTAATCAGAATGTCCTTCTGATGTCCCCCCAGCCTCTGACTCAGGG TCCCAGGCTGGCTTCTCTCCCTACTCCATTGAACTCTGAGCCAGGAA GCCCTCTATTGGATGATGCAAAGGTCAAAGATGAGCCAGACTCTCCTCCTGTGGCGCTGGGCATGGTAGACCGCTCACGGATTCTTCTGTGTGTCCTCACCTTCCTGTGCCTCTCCTTTACCCCCTGACTTCCCTGCTGCAGTGGGGAGGGGGCCCACGACTCTGACCAGCACCCACACTCAGGCTCTGGCCGCAGTGTCCTGTCATTCGAGTCAGGTTCTGGGGGGCTGGTTTGACTGGATGATGCCTACTCTTCTCTTATGGCTGGTAAATGGTGTGATTGTCCTGAGCGTCTTTGTGAAGCTGCTGGTTCATGGGGAGCCAGTGATCCGGCCACACTCGCGCTCCTCGGTCACCTTCTGGAGGCACCGGAAACAGGCAGATCTGGATCTCGCCAGAGGAGATTTTGCAGCTGCTGCCGGCAACCTACAAACCTGCCTGGCAGTTTTGGGCCGGGCACTGCCCACCTCCCGCCTGGACCTGGCCTGCAGCCTCTCCTGGAACGTGATCCGCTACAGCCTGCAGAAGCTACGCCTGGTGCGCTGGCTGCTCAAGAAAGTCTTCCAGTGCCGGCGGGCCCACGCCAGCCACTGAGGCAGGCTTTGAAGACGAAGCTAAGACCAGCGCCCGGGATGCGGCTCTGGCCTATCACCGGCTGCACCAGCTGCACATCACAGGGAAGCTTCCTGAGGATCCGCCTGTTCCGATGTACACATGGCGTTGTGTGCCGTGAACCTGGCTGAATGTGCAGAGAGAGAAGATCCCACCGAGCACACTGGTTGAGATCCATCTGACTGCTGCCATGGGGCTCAGACCCGGTGTGGAGGCAAGCTGGGCTTCCTGGCCAGCTACTTCCTCAGCCGAGCCCAGAGCCTGTGTGGCCCCGAGCACAGTGCTGTTCCTGACTCCCTGCGCTGGCTCTGCCACCCCCTGGGCCAGAAGTTTTTCATGGAGCGGAGCTGGTCTGTGAAGTCAGCTGCCAAGGAGAGTCTATACTGTGCCCAGAGGAACCCAGCTGACCCCATTGCGCAGGTCCACCAGGCCTTCTGCAAGAACCTGCTGGAGCGAGCTATAGAGTCCTTGGTGAAACCTCAGGCCAAGAAGAAGGCTGGAGACCAGGAAGAAGAGAGCTGTGAATTCTCCAGTGCTCTGGAGTACTTGAAATTACTTCATTCTTTTGTGGACTCTGTGGGGGTTATGAGCCCCCCACTCTCCAGGAGCTCCGTGCTCAAGTCCGCCCTGGGTCCAGACATCATCTGTCGGTGGTGGACGTCTGCAATCACTGTGGCCATCAGCTGGCTCCAGGGAGACGATGCAGCTGTGCGCTCTCATTTTACCAAAGTGGAACGCATCCCCAAGGCCTGGAAGTGACAGAGAGCCCCCTGGTGAAGGCCATCTTCCATGCCTGCAGAGCCATGCATGCCTCACTCCCTGGGAAAGCAGATGGGCAGCAGAGTTCCTTCTGCCATTGCGAGAGGGCCAGTGGCCACCTATGGAGCAGCCTCAACGTCAGTGGGGCCCACCTCTGACCCTGCCCTCAACCACGTGGTCCAGCTGCTCACCTGTGACCTGCTACTGTCGCTACGGACAGCGCCTGGCAAAAACAGGCCAGTGCCAGCAGGCTGTGGGGGAGACCTACCACGCGTCAGGCGCTGAACTGGCGGGCTTCCAACGGGACCTGGGCAGCCTGCGCAGGCTGGCACACAGCTTCCGCCCAGCATACCGCAAGGTGTTCCTG CATGAAGCCACCGTGCGCCTGATG GCAGGAGCCAGCCCCACCCGCACCCACCAGCTGCTGG AACACAGCCTGCGGCGGCGCACCACGCAGAGCACCA AGCACGGAGAGGTGGATGCCTGGCCC GGCCAGCGAGAGCGGGCCCACCGCCATCCTGCTGGCCTGCCGCCACCTGCCCCTCTCCTTCCTCTCCTCCCCGGGCCAGCGGGCAGTGCTGCTGGCCGAAGCTGCCCGCACCCTGGAGAAGGTGGGCGACCGGCGCTCCTGCAACGACTGCCAGCAGATGATTGTTAAGCTGGGTGGTGGCACTGCCATTGCCGCCTCCTGACCACCAGGCTCAGCCCACCCCTCCACCTCTCTCTCGATTTCTCTCTCTCCCCCTCAGCATCTTCCC GCTGAGAGTGGTGGGGAAGAGCCTTGTCTTCTTAGCTGTCACCTGCCGAGGCTTCTGGGCCACTCAGGCCAGTGCACCCCTGGGCAGAGCCCCTTAAAGCTGCTGTCACTAGATGCCCATGGTCCAGGGCCTGGTGGGCGTGAGAGGATAGGTGGGCAGGGCAGAAACTGGGCAGCCCTGACTTGATAGCAGCAGGGGGAGCTCCCAAGCTGCCAAGCCCCCTGCCTCCAGCCTTCCTGAGTTTCTCTCTCCTGAACCCTACTCTCTCCTTTTTGCTTCCTCAGTTTTTATCAGGCTTTCTCTGGGGGACAGCAGTCTCTGAGCACCAGGGAGCAGTTGCCCTCAGGCCTGTGCCCAGCATGCCCTCCCCTTTTTATACGAATGTTTTCTACCAGTGTGCTTGGGGTTTGCCATGATGCGAGGCTGAGTTGCTGTAGCGTCTTGATTCTCTCCCTGGGTCTGCGTTCCCTCCCCTGGGCCTGACTGAGCCTGCTCATTGTTTTTCCCTTTATTACACAGGACAGCCAGGGGAGGAGGGGGCCCAGCCCTGGGA GGCTGGTGGGAGGCAGGGGG CAGGCCTGCGGATGCATGAAATAATGTTGG CATTATTTTTTAATTTTTTAAAAAATAaatGGTA TCTTATTTAATTGTCCTGTTCCTTCCCACTCCCCGCC TCCTAGGATGTTAGCCCAAGCTCAGGGTAGGCCCAGGGGGCTGGGAGAAAATGAAGCCACCCATGGGGACTGGGGACCAGGGGCCTTCAGCATGGCTTCTAGGTTCCCTCCTCCCCCTACCCCATCTCCTACCTCCACAGTACAGACTGTCCCAACTTAACAGTGGTTCAACTTAAACCATGTTTCAACTTTACAATTGGTCTGTTGGGGTATTAAATGAATTTGTGACTTAGGATATTTTCATTTATGATGGGTTTATCAGGAAGTAACCCCATGGTAAGTGAGGCATATCTGTATATATTTAAACCTAATTAATTCTTGAGCTGAAAATAAATAAACCAGGATGGCAGGGACCAACCCCTAATCCCTCCCCAGCGGCAAGCCCCTCTTTCAGAGTGGGCAGAGGGTTGCCTATGGTGGGCACTAGGAATGAGGTCCCCTGCCTCGATGCGGGTCCTAGGAGAAAAAGTCCTACTTTTCTGGGTCCCCAGGTGCAGCACCTCCCGGAGACTGTTTCTCCCATGGCCTCCTGAGTGATGGGCCCTGCCTCCCTGTGCCTCATCCTCAGGCTGGTTGGAGCAGAGGGTGGGCAGGAGCCCCAGCACAGACTGGGGGGTGCTCACAGCAGGGCCACCTTGATGCAGGCTGGAATGTTATCCTGGGGTGTGCTTGGACCCCACCTGCTTTCTTTCTCTCCTGCCCCTCCCCTACTCTCACTGTAATTTATGGACCCTGCCCGCCTGCGTGTTGTGTGTATGTCCTGTGCCTTTCTCACTATTGTTTTGGTGTGGGAGGGGGTGGTTTTTCACTGAAAAGGGGGGTACACCTATAGCTTTCTTGATGTTCAATCAATCAGTCACTGTGTCCCAGACATATTCAATAAACACAGATTGG TACCACCCA

**S1.4** HBx and SREBP2 dual-luciferase reporter gene plasmid

HBx

ATGGCTGCTAGGCTGTGCTGCCAACTGGATCCTGCGCGGGACGTCCTTTGTTTACGTCCCGTCGGCGCTGAATCCTGCGGACGACCCTTCTCGGGGTCGCTTGGGACTCTCTCGTCCCCTTCTCCGTCTGCCGTTCCGACCGACCACGGGGCGCACCTCTCTTTACGCGGACTCCCCGTCTGTGCCTTCTCATCTGCCGGACCGTGTGCACTTCGCTTCACCTCTGCACGTCGCATGGAGACCACCGTGAACGCCCACCAAATATTGCCCAAGGTCTTACATAAGAGGACTCTTGGACTCTCAGCAATGTCAACGACCGACCTTGAGGCATACTTCAAAGACTGTTTGTTTAAAGACTGGGAGGAGTTGGGGGAGGAGATTAGGTTAAAGGTCTTTGTACTAGGAGGCTGTAGGCATAAATTGGTCTGCGCACCAGCACCATGCAACTTTTTCACCTCTGCCTAA

SREBP2 dual-luciferase reporter gene plasmid

Vector：MCS-firefly_Luciferase-PloyA-Tk-Renilla_Luciferase-PolyA

SREBF2 NM_004599-promoter:

TTTCACCAAGCTGGCCAAGCTGGTCTCGAACTCCCGACCTCAGGCAATCCGCCCACCTCAGCCTCCCAAAGTGCTGGGATTACAGGCGTGAGCCACCTTGCCCGGACTCTCTTTGTATTTTTTCTTTCTTTTTTTTGAGACAGAGTCTTGCTCTGTCTCCCAGGCTGGAGTGCAGCGACATGATCTCAGCTCACTGCAGCCTCTATCTCCCAGGTTCCGGCAGTTCTCCTACCGGTTAGCTGGGATTACAGGCATGCGCCACCACACCCGGCTAATTTTTGTATTTTTAGTAGAGACGGGGTTTCACCATGTTGGTCAGGCTGGTCTCCAACTCCTGACCTCAAATGATCTGCACACCTCGGCCTCCTAAAGTGCTGGAATTACCGGTGTGAGCCACCGCGCCTGGCCAGATCCTTATTTTTTCAATTGCAATACAGCAGTATGCAAGGCACTTACTGTATGCCAAAGCTTATGCCGAGAGCTGGCTGCCAGCAGCTCCTCCTGCCTCGAGGCCTTTGTCCATGTCATCATTTCCCCTTTGGCTGTAACAGCTGCAGTTCTCTCATAATTTTAGGTTTCATCTTAAATGTCACTTCTTCAGAGGCTTCTTGACAGCACAAGTCAGGGCCCTTGTTAGTTTCATCACATCCATTACCTTTCCTTGATCACTTGTACCATCATCTGTAATTTATTTTTATGTTTAATACGGGTGTGGGATTTCTGATTTTTTTTTTTGCTAGACTGGAAGCTCTCAGAGGGCAGGAACCATTTGTTTCCCTGCCACAGAGATGTTCAATAAATATTGTCAATTGAATGTGCAAATAATCTCACTTAACCACAGCTATGAGGGATGCTATTTTATCCCCATTTTAGAGATGACTAAACTGAGGCTTAGAACAATTAACCCAAGGTCACAGTGAATTAGTGGATCCATGTGTCTGCCTCCACACAGCCTGTGTGTTCTTAATTATTGATCACTAAGCAACAGCTGCCTCTCACAAACTGGCAGGAAACTTCAACTTTTCCCTTTTATCAATATATTGTGAACGCCTTTTCTTGCTAATAAATATACATTTGTGTGCGTAATTTTTAATGATCCCATGGTATTCCATCGTGTGGATGTAATTTACATATTCATTTCCTTATTCTCACTTCTTCCTAGTTACAGCACTTCTTTAAGCACTTGTAATTTTTATTCCTTTGGATAAATTCCTAGAAGTTTACAACACGACCAGCTGCAAGATGGGGCAGTGAGGTGCTTGAAGGAGTGGGTGGGCTGCACTTCAGAATCCCGGTGGAACTTTTTCAAAAAACTCGTGCCCGGGGCCCCCCTCTCAGGAGGCCGTCGGTGGGCCTGGGCACTGGCGTGGTTTTCAGGTCCTCAGTGGTTCCGAGGTGCCAGAGATTGAGGACCACTGGAGTAAGCGCGAGGTCACGGCGGGTGCAAAGCAGAAGACGTAAAATCCTGACCGCCCTGGGACAATCTGCAACCTTGTCAAGCCTCCGTGCCCGCCTTTGTAAGGAGGCTGGGAGAAGCGGGACTTGGATGACCCGGACTCCCGTCCCGAGCGCTGGGGTCCAGGTTTGACTCCGCTGGCTTTCTCAGGCGGGCAGGGCGGGGGTAGGCAGCTGGGAAGATGACGTAATGTGCTCCCAGCCAGGCCTGGAGGCGGCCAGCGGTCGCAGGTGGAGGGTGGCCTGTTAACCCTTCACTCCCAGGCCAGTGGACGGACTTGCGTGTGGCGCAGATCCAACGGAGAAGGCAGCGGCTCCTTTAAACAAGGCGGAGAAGGTTAAGATGATGACCGGACGGCTACTCCAGGCATTCGCTCCGAGGCCGCGGGGGGAGGGACCTCACTATGCAAATCTGAGCTGCTGATCGATGACGCGCCATCACCCCACGCACCGCTTCGCTCGCCCATTGGCTGAGATGAGCCTGGTCCCATTGACAACAAACAGGGGGGCGCGCGGCCTGGAGGCGGGGCCGCAGGGGGCGCGGGCTGGGGCGGGGGAATCCCGCCCCGCCCTTTCTGTGCGG
